# Navigating the Labyrinth of Hepatocellular Carcinoma: Leveraging AI/ML for Precision Oncology

**DOI:** 10.32604/or.2026.074185

**Published:** 2026-04-22

**Authors:** Abdul Manan, Sidra Ilyas

**Affiliations:** 1 Department of Molecular Science and Technology, Ajou University, Suwon, Republic of Korea; 2 Department of Herbal Pharmacology, College of Korean Medicine, Gachon University, 1342 Seongnamdaero, Sujeong-gu, Seongnam-si, Republic of Korea

**Keywords:** Hepatocellular carcinoma, immunotherapies, transcriptomic, multi-omics, artificial intelligence, machine learning

## Abstract

Hepatocellular carcinoma (HCC) remains a significant global health challenge, with therapeutic efficacy in advanced stages often limited by underlying liver dysfunction and adaptive resistance. In this review, the evolving landscape of molecular targets and combinatorial strategies is critically examined, with a particular focus on the transition from preclinical discovery to clinical application. While traditional molecular heterogeneity is acknowledged, the aim is to elucidate how emerging computational paradigms are redefining target discovery and therapeutic stratification in HCC. The primary purpose is to evaluate the role of Artificial Intelligence (AI) and Machine Learning (ML) as integrative tools for translating high-dimensional multi-omics data into clinically actionable insights for HCC management. Special attention is given to the capacity of AI-driven frameworks to analyze complex datasets derived from genomics, transcriptomics, proteomics, metabolomics, and epigenomics, thereby enabling the identification of novel predictive biomarkers, patient subgroups, and rational drug combinations. By synthesizing recent preclinical and clinical evidence, this review highlights how AI-guided approaches can accelerate biomarker validation and optimize therapeutic decision-making. Furthermore, the convergence of AI with spatial transcriptomics, digital pathology, and single-cell technologies is discussed as a transformative infrastructure for decoding tumor–microenvironment interactions and spatial heterogeneity. These integrative strategies provide unprecedented resolution into tumor evolution, immune landscapes, and resistance mechanisms. Collectively, the evidence reviewed supports the conclusion that AI-enabled, multi-omics–driven approaches are instrumental in advancing HCC treatment toward a new era of adaptive, spatially informed, and precision-based personalized medicine.

## 1 Introduction

Hepatocellular carcinoma (HCC) represents a formidable public health challenge, constituting the third leading cause of cancer related mortality globally. With an estimated 906,000 new diagnoses and 830,000 deaths in 2022 alone, its incidence is projected to rise by 55% by 2040, underscoring the urgent need for transformative therapeutic strategies [1,2]. Accounting for over 80% of all liver cancer cases, HCC is frequently diagnosed at intermediate or advanced stages, resulting in limited curative options and poor long-term survival. HCC is a biologically and clinically heterogeneous disease arising from diverse etiologies, including chronic hepatitis B virus (HBV) and hepatitis C virus (HCV) infections, alcohol-associated liver disease, metabolic dysfunction-associated steatotic liver disease (MASLD/NASH), and environmental carcinogens such as aflatoxin. These etiological factors shape distinct molecular, immunological, and metabolic tumor landscapes, contributing to substantial inter- and intra-tumoral heterogeneity [3]. This heterogeneity underlies variable therapeutic responses, frequent treatment resistance, and high recurrence rates, posing major challenges for effective disease management.

Despite remarkable progress in surveillance, diagnostic modalities, and systemic therapy, clinical management of advanced HCC remains suboptimal. Multi-kinase inhibitors, anti-angiogenic agents, and immune checkpoint inhibitors (ICIs) have modestly extended survival in select patient populations; however, intrinsic and acquired drug resistance, profound tumor heterogeneity, and the underlying fragility of diseased livers continue to restrict durable outcomes. Extensive genomic profiling has catalogued recurrent mutations in canonical drivers and chromatin remodeling, yet the functional interplay of these alterations and their ultimate impact on therapeutic vulnerability remain incompletely understood.

Precision oncology has therefore emerged as a critical framework for advancing HCC treatment by aligning therapeutic decisions with tumor-specific molecular and biological characteristics. Unlike conventional staging systems, precision approaches aim to integrate molecular alterations, TME features, and host factors to enable individualized therapy selection. However, the clinical implementation of precision medicine in HCC is challenged by limited tumor tissue availability, spatial and temporal heterogeneity, and the complexity of interpreting high-dimensional biological data [4]. Recent advances in high-throughput multi-omics technologies including genomics, transcriptomics, proteomics, epigenomics, and metabolomics have provided unprecedented insights into the molecular architecture of HCC [5]. When combined with spatial transcriptomics, these approaches allow detailed characterization of tumor microenvironment (TME) interactions and immune landscapes. Nevertheless, the scale and complexity of these datasets exceed the capacity of conventional analytical methods.

Artificial intelligence (AI) and machine learning (ML) have consequently emerged as indispensable tools for integrating multi-modal data and extracting clinically actionable insights [6]. In HCC, AI-driven models are increasingly applied to radiomics, digital pathology, multi-omics integration, prognostic modeling, and therapeutic response prediction [5,7]. These approaches hold promise for improving early diagnosis, refining risk stratification, guiding treatment selection, and enabling adaptive therapeutic strategies. This review synthesizes current advances in HCC treatment through the lens of precision oncology, with a particular focus on the integration of multi-omics profiling, spatial transcriptomics, and AI-driven computational models. We critically examine emerging molecular targets, combination therapeutic strategies, and AI-enabled clinical decision support systems, while highlighting translational challenges, unmet needs, and future directions for personalized HCC management.

## 2 Overcoming Therapeutic Limitations: Tumor Heterogeneity and Drug Resistance

The biological complexity of HCC presents formidable obstacles to effective therapeutic intervention. Two intertwined challenges are profound tumor heterogeneity and the rapid emergence of drug resistance, which account for the historically limited success of systemic therapies in advanced disease.

### 2.1 Tumor Heterogeneity as a Central Barrier

HCC is characterized by inter-patient heterogeneity arising from diverse etiological factors leaving distinct mutational and epigenetic imprints [8,9]. Host specific variables, including gut microbiome, immune landscape, and germline background, add additional layers of heterogeneity [10]. At the molecular level, oxidative stress, recurrent alteration in oncogenes (*CTNNB1*, *c-Myc*, *c-Met*, *TERT*, *PIK3CA*), tumor suppressors (*AXIN*, *RB*, *PTEN*, *TP53*, *KEAP1*), and chromatin regulators (*ARID1A*, *ARID2*), drive distinct phenotypes, such as the “proliferation” vs. “non-proliferation” subclasses, each carrying unique prognostic implications [11,12].

**Figure 1:**
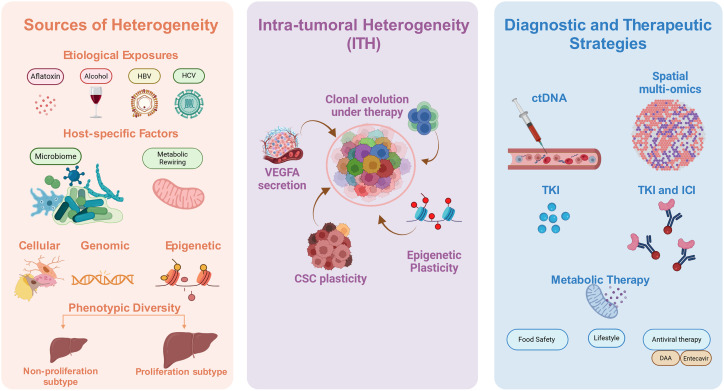
Schematic representation of the drivers and consequences of tumor heterogeneity in hepatocellular carcinoma (HCC). Exposures (HBV/HCV, alcohol, MASLD/NASH, aflatoxin) and host specific factors (immune system, microbiome, germline variants) converge with tumor intrinsic alterations (mutations, epigenetic changes, metabolic rewiring) to generate profound inter-patient and intra-tumoral heterogeneity. This diversity fuels clonal evolution, adaptive resistance, and immune evasion, ultimately limiting the efficacy of targeted therapies and immunotherapies. Emerging strategies such as liquid biopsy, spatial multi-omics, and adaptive combination therapies may offer the potential to convert descriptive molecular classification into actionable stratification, guiding dynamic and personalized treatment for HCC. HCC: Hepatocellular Carcinoma; HBV: Hepatitis B Virus; HCV: Hepatitis C Virus; MASLD: Metabolic Dysfunction-Associated Steatotic Liver Disease; NASH: Nonalcoholic Steatohepatitis; TKI: Tyrosine Kinase Inhibitor; ICI: Immune Checkpoint Inhibitor.

Equally critical is intra-tumoral heterogeneity (ITH), reflecting the coexistence of genetically and phenotypically distinct subclones within individual tumors. ITH drives clonal evolution, metastasis, recurrence, and therapeutic resistance [13]. Under therapeutic pressure, resistant subclones with survival advantages expand a Darwinian process that underlies the modest benefits of multi-kinase inhibitors and fosters multi-drug resistance. Beyond genetic diversity, ITH drives functional divergence, including differences in proliferative capacity, invasiveness, angiogenic signaling, and immune evasion (Fig. 1). Clinically, this diversity complicates tumor sampling, as a single biopsy rarely captures the complete genomic and immune architecture of a tumor, necessitating multi-regional or liquid biopsy approaches, which remain technically and clinically challenging [14].

### 2.2 Towards Adaptive Therapeutic Strategies and Actionable Stratification

**Figure 2:**
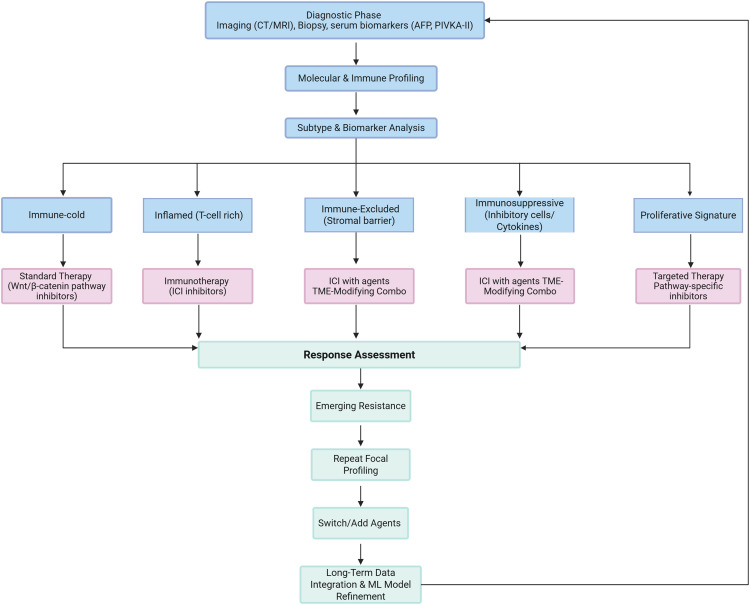
Dynamic decision tree for personalized HCC management. The workflow emphasizes a personalized, adaptive, and systems level approach, aiming to translate molecular understanding into actionable clinical strategies, ultimately overcoming heterogeneity and resistance in HCC. CT: Computed Tomography; MRI; Magnetic Resonance Imaging; AFP: Alpha-fetoprotein; PIVKA-II: Protein Induced by Vitamin K Antagonist-II; ICI: Immune Checkpoint Inhibitor; TME: Tumor Microenvironment; ML: Machine Learning; HCC: Hepatocellular Carcinoma.

Addressing this biological reality requires a shift from static to adaptive therapeutic strategies. Rational combinations—whether simultaneous or sequential—must target multiple vulnerabilities to prevent clonal escape. While recent molecular classifications have categorized HCC by oncogenic pathways (e.g., Wnt/β-catenin) and immune phenotypes (e.g., “inflamed” vs. “excluded”), these frameworks remain largely descriptive. To achieve clinical impact, they must evolve into actionable stratification models that link subtypes to specific biomarkers and therapeutic vulnerabilities. An ideal clinical workflow functions as a dynamic decision tree (Fig. 2), where longitudinal monitoring via liquid biopsies and spatial multi-omics guides real-time adjustments in treatment, shifting from empirical selection to mechanism-based precision medicine [13].

### 2.3 Mechanisms of Intrinsic and Acquired Drug Resistance in HCC

HCC frequently exhibits insensitivity to conventional chemotherapy agents and a propensity to develop multidrug resistance (MDR) rapidly during treatment, leading to reduced survival and poor prognosis. This resistance arises from a complex interplay of molecular and cellular mechanisms (Table 1). Overexpression of ATP-binding cassette (ABC) transporter proteins such as P-glycoprotein (P-gp/ABCB1), Breast Cancer Resistance Protein (BCRP/ABCG2), and multidrug resistance-associated proteins (MRPs/ABCC), actively pump anticancer drugs (adriamycin, paclitaxel, 5-FU, sorafenib) out of tumor cells, thereby diminishing the drug’s therapeutic efficacy and inducing MDR [15].

**Table 1:** Major sources of tumor heterogeneity in HCC.

Category	Specific Features	Representative Agents	Functional Implications
Etiological drivers	Viral infections, metabolic disorders, toxins	HBV, HCV, MASLD/NASH, alcohol, aflatoxin	Shape mutational spectrum, regional prevalence
Genetic alterations	Driver mutations, chromosomal instability, copy number variation	*TERT*, *TP53*, *CTNNB1*, *ARID1A*, *KMT2C*	Define molecular subclasses, influence therapy response
Epigenetic regulation	DNA methylation, histone modifications, non-coding RNAs	Hypermethylation, histone acetylation, miRNAs/lncRNAs	Drive gene expression variability, lineage plasticity
Immune microenvironment	Differential immune infiltration, checkpoint expression, cytokine signaling	CD8^+^ T cell density, PD-L1 expression, VEGFA secretion	Immune evasion, variable immunotherapy response
Cancer stem cells (CSCs)	Phenotypic plasticity, self-renewal, epithelial mesenchymal transition (EMT)	EpCAM, CD133, ALDH subpopulations	Tumor initiation, recurrence, resistance
Microenviron- mental cues	Stromal composition, angiogenesis, hypoxia, extracellular matrix remodeling	VEGFA driven angiogenesis, Cancer-associated fibroblasts (CAF), hypoxic niches, ECM stiffness	Promote invasion, metastasis, immunosuppression
Host factors	Germline variants, gut microbiome, sex related biology	SNPs in drug metabolism genes, microbiota composition	Inter-patient heterogeneity, variable drug metabolism

Note: HBV: Hepatitis B Virus; HCV: Hepatitis C Virus; MASLD: Metabolic Dysfunction-Associated Steatotic Liver Disease; NASH: Nonalcoholic Steatohepatitis; TERT: Telomerase Reverse Transcriptase; TP53: Tumor Protein p53; CTNNB1: Catenin Beta 1; ARID1A: AT-Rich Interactive Domain-Containing Protein 1A; KMT2C: Lysine Methyltransferase 2C; miRNAs: MicroRNAs; lncRNAs: Long Non-Coding RNAs; CD8^+^: Cluster of Differentiation 8–Positive T Cells; PD-L1: Programmed Death-Ligand 1; VEGFA: Vascular Endothelial Growth Factor A; CSCs: Cancer Stem Cells; EMT: Epithelial–Mesenchymal Transition; EpCAM: Epithelial Cell Adhesion Molecule; CD133: Cluster of Differentiation 133; ALDH: Aldehyde Dehydrogenase; CAF: Cancer-Associated Fibroblasts; ECM: Extracellular Matrix; SNPs: Single-Nucleotide Polymorphisms.

Dysregulation of programmed cell death (PCD) pathways is a hallmark of MDR. This includes *TP53* mutations and the upregulation of anti-apoptotic proteins like Bcl-2 and MCL-1 [16]. PANoptosis is an emerging inflammatory PCD pathway that integrates molecular features of pyroptosis, apoptosis, and necroptosis through the formation of multi-protein PANoptosome complexes. Disruption of PANoptosis related genes can influence tumor progression, immune evasion, and therapy resistance in multiple malignancies, and that PANoptosis linked signatures have potential as biomarkers of prognosis and immunotherapeutic responsiveness in HCC [17]. Furthermore, enhanced DNA repair capabilities (e.g., via XRCC4-like factor in the non-homologous end-joining (NHEJ) pathway allow HCC cells to survive the genomic stress induced by conventional cytotoxic agents [18].

Reversible epigenetic modifications can silence tumor suppressors or activate pro-survival genes. Similarly, while basal autophagy is suppressive, activated autophagy in established tumors helps cells survive metabolic and therapeutic stress.

TME is a complex ecosystem comprising various normal cells (e.g., fibroblasts, endothelial cells, immune cells) and the extracellular matrix (ECM), acts as a protective niche. Specific metabolites (e.g., 27-hydroxycholesterol) or ECM stiffness can impede drug delivery and foster a pro-survival signalling. Furthermore, cancer stem cells (CSCs) (marked by EpCAM or CD133) contribute to recurrence and metastasis due to their inherent resistance and capacity for dormancy (Table 2).

**Table 2:** Mechanisms of drug resistance in HCC and strategies to overcome them.

Category	Resistance Pathway/Mechanism	Impact on Resistance (Phenotype)	Mechanistic/Immuno-Metabolic Insights	Therapeutic Strategy to Overcome	Ref.
I. Cellular Stress & Survival	Dysregulated Apoptosis: *TP53* loss/mutation, BCL-2/MCL-1 overexpression	Malignant Clonal Survival: Suppresses programmed cell death; confers resistance to cytotoxic agents and TKI	N/A (Direct survival pathway)	TP53 reactivators, BH3-mimetics, dual BCL-2/MCL-1 inhibition	[19]
	Activated Autophagy: ATG, Beclin-1 mediated	Drug Tolerance/Adaptation: Cytoprotective autophagy enables metabolic adaptation under therapeutic stress	Supports energy production (ATP), recycles organelles to maintain proliferation and survival	Autophagy inhibitors (HCQ, CQ) combined with systemic therapy (e.g., TKIs)	[20,21]
	ER Stress/UPR Activation: (BiP/GRP78, PERK, IRE1α, ATF6)	Survival under Proteotoxic Stress: Promotes cell survival, cross talks with autophagy and apoptosis pathways	Alters redox balance, links closely with glycolysis and lipid metabolism for protein folding capacity	Target PERK/IRE1, or combine ER stress modulators with targeted/pathway inhibitors	[22,23]
	CTNNB1 (β-catenin), Wnt Pathway Activation	Immune Exclusion & Proliferation: Drives HCC proliferation, promotes immune evasion, and general therapy resistance	Immuno-Metabolic Link: Alters chemokine/cytokine profiles to exclude CD8^+^ T cells; promotes metabolic adaptation via upregulation of glycolysis and glutaminolysis	β-catenin-TCF/LEF inhibitors, combination with ICIs, targeted metabolic inhibitors	[24]
II. Genetic & Oncogenic Signalling	ARID1A Loss-of-Function	Chromatin Disruption & EMT: Leads to Epithelial-Mesenchymal Transition (EMT), metastasis, and broad therapy resistance	Metabolic Rewiring: Upregulates glycolysis and glutamine/glutathione metabolism to support proliferation and critical redox homeostasis	Epigenetic modulators (HDAC/BET inhibitors), metabolic pathway inhibitors, synthetic lethal strategies	[25]
	PI3K/Akt/mTOR, MAPK/ERK, FGFR4 Activation	Proliferation & Survival Signaling: Provides strong survival signals and drives uncontrolled cell division; supports angiogenesis	Alters glucose uptake, protein/lipid biosynthesis; directly supports pro-survival pathways.	Small molecule inhibitors (mTOR, MEK, FGFR inhibitors), rational combination therapies	[26]
III. Tumor Microenvironment & Immunosuppression	Immunosuppr- essive TME: (VEGFA, Tregs, MDSCs, TAMs, CAFs)	ICI Resistance/Inefficacy: Inhibits anti-tumor immunity; reduces ICI efficacy; promotes angiogenesis and hypoxic niches	Hypoxia/Metabolism Driven: Hypoxia driven metabolic rewiring favors immune suppression; VEGF signaling affects endothelial barrier function and T cell infiltration	Combination Therapy: Anti-VEGF and ICIs (e.g., Atezolizumab/Bevacizumab), Treg/MDSC reprogramming, vascular normalization	[27]
	Immune Checkpoint Upregulation: PD−1/PD−L1, CTLA−4, TIM−3/LAG−3	Immune Evasion: Leads to T cell exhaustion and profound immunosuppression	N/A (Direct T cell inhibitory pathways)	ICIs: Combinations of Anti-PD−1/L1 and Anti-CTLA−4 (e.g., Durvalumab and Tremelimumab), novel combinatorial checkpoint blockade	[28]
IV. Metabolic Vulnerabilities	Enhanced Glycolysis (Warburg Effect): (GLUT1, HK2, PKM2, LDHA)	Proliferation & Survival: Supports rapid cell growth and survival under harsh TME conditions	Drives Immuno-Metabolism: Generates lactate, which promotes lactylation and drives an immunosuppressive TME by supporting Tregs and MDSCs	Glycolytic inhibitors (2-DG, PKM2 inhibitors), combination with systemic or immunotherapy	[29,30]
	Arginine Auxotrophy: ASS1/OTC deficiency	Metabolic Dependency: Tumor cells become dependent on exogenous arginine supply for survival	Limits *de novo* arginine synthesis; tumor relies entirely on extracellular supply.	Arginine-degrading enzymes (ADI-PEG-20, rhArg1/BCT100)	[31]
V. Epigenetic & Non-coding RNA Dysfunction	HDAC/DNMTs Upregulation	Chromatin Alterations: Leads to altered gene expression, promoting therapy resistance and EMT	Epigenetic modulation of metabolic genes (glycolysis, glutaminolysis) and immune related genes (HLA expression)	HDAC inhibitors (Panobinostat, Chidamide), DNMT inhibitors (Guadecitabine); synergy with immunotherapy	[32]

Note: ATP: Adenosine Triphosphate; ADI-PEG-20: Pegylated Arginine Deiminase; ARID1A: AT-Rich Interactive Domain-Containing Protein 1A; ASS1: Argininosuccinate Synthetase 1; ATF6: Activating Transcription Factor 6; ATG: Autophagy-Related Genes; Beclin-1: Beclin-1 Autophagy Initiation Protein; BCL-2: B-cell Lymphoma 2; BET: Bromodomain and Extra-Terminal Domain Proteins; BiP: Binding Immunoglobulin Protein; BCT100: Recombinant Human Arginase-1; CAFs: Cancer-Associated Fibroblasts; CQ: Chloroquine; CTLA-4: Cytotoxic T-Lymphocyte-Associated Protein 4; CTNNB1: Catenin Beta 1; DNMTs: DNA Methyltransferases; EMT: Epithelial-Mesenchymal Transition; ERK: Extracellular Signal-Regulated Kinase; FGFR4: Fibroblast Growth Factor Receptor 4; GLUT1: Glucose Transporter Type 1; GRP78: Glucose-Regulated Protein 78; HCQ: Hydroxychloroquine; HDAC: Histone Deacetylase; HK2: Hexokinase 2; HLA: Human Leukocyte Antigen; ICI: Immune Checkpoint Inhibitor; IRE1α: Inositol-Requiring Enzyme 1 Alpha; LAG-3: Lymphocyte-Activation Gene 3; LDHA: Lactate Dehydrogenase A; LEF: Lymphoid Enhancer-Binding Factor; MAPK: Mitogen-Activated Protein Kinase; MCL-1: Myeloid Cell Leukemia-1; MDSCs: Myeloid-Derived Suppressor Cells; mTOR: Mechanistic Target of Rapamycin; OTC: Ornithine Transcarbamylase; PD-1: Programmed Cell Death Protein 1; PD-L1: Programmed Death-Ligand 1; PERK: Protein Kinase RNA-like Endoplasmic Reticulum Kinase; PI3K: Phosphoinositide 3-Kinase; PKM2: Pyruvate Kinase Muscle Isoform 2; rhArg1: Recombinant Human Arginase-1; TAMs: Tumor-Associated Macrophages; TCF: T-Cell Factor; TIM-3: T-Cell Immunoglobulin and Mucin-Domain Containing-3; TKI: Tyrosine Kinase Inhibitor; TME: Tumor Microenvironment; TP53: Tumor Protein p53; Tregs: Regulatory T Cells; UPR: Unfolded Protein Response; VEGFA: Vascular Endothelial Growth Factor A; β-catenin: Beta-Catenin; 2-DG: 2-Deoxy-D-Glucose.

These resistance mechanisms rarely operate in isolation; they form a redundant network where targeting a single node often triggers compensatory activation of another. For example, epigenetic changes may upregulate ABC transporters, while an immunosuppressive TME protects resistant CSCs. This inherent redundancy necessitates multi-target combination therapies designed to disrupt several nodes concurrently. By identifying master regulators or common vulnerabilities (Table 2), clinicians can move toward highly personalized, mechanism-based strategies that delay resistance and improve long-term outcomes.

## 3 Molecular Drivers and Therapeutic Targets in HCC

The complex landscape of HCC, marked by significant heterogeneity and drug resistance, necessitates the identification of novel molecular targets and the exploitation of therapeutic vulnerabilities to develop interventions that are more effective. Research efforts are increasingly focused on unraveling the intricate oncogenic signaling pathways and identifying unique tumor-associated antigens and targets (Table 3). Major pathways and molecular mechanisms implicated in HCC include:

**Table 3:** The mechanism of action of novel molecular targets in HCC development.

Pathway	Molecular Targets	Role in HCC Pathogenesis	Therapeutic Implication	Refs.
Oncogenic signaling & cell survival	Folate Receptor α (FRα), TP53, FGFR2, HGF/c-MET, Akt, Cyclin D1	Abbreant growth signaling, impaired apoptosis, and sustained proliferative capacity drive tumor initiation and progression	Targeted inhibitors, gene restoration strategies, and cell-cycle blockade to suppress tumor growth	[42–48]
Angiogenesis & hypoxia adaptation	VEGF, HGF/c-MET	Promotes neovascularization, hypoxic adaptation, and resistance to systemic therapies	Anti-angiogenic agents and MET inhibitors to restrict tumor vascular supply	[46,47,49,50]
Immune evasion & immunosuppression	PD-L1, TGF-β	Suppression of cytotoxic T-cell activity and immune exclusion within the TME	Immune checkpoint blockade and TGF-β pathway inhibition to restore anti-tumor immunity	[51–54]
Developmental & stemness signaling	Wnt/β-catenin pathway, GPC3	Maintenance of stem-like features, EMT, invasion, and chemoresistance	Targeting Wnt signaling and GPC3-directed immunotherapies to limit tumor aggressiveness	[55,56]
Metabolic reprogramming	AMPK, HK2	Warburg metabolism and altered energy sensing support rapid tumor growth and survival under stress	Metabolic modulators and glycolysis inhibitors to disrupt tumor bioenergetics	[57,58]
Epigenetic & RNA modification control	NSUN2, METTL3, IGF2BP1, PRMT5, HDACs, Histone lactylation	Stabilization of oncogenic transcripts, chromatin remodeling, and transcriptional plasticity promote tumor progression	Epigenetic drugs and RNA-modifying enzyme inhibitors to reprogram malignant gene expression	[59–62]
Non-coding RNA regulation	lncRNAs (HOTAIR, MALAT1), miRNAs (miR-122, miR-21)	Post-transcriptional regulation of oncogenes and tumor suppressors drives invasion and therapy resistance	RNA-based therapeutics (mimics, antagomirs) to modulate irregular gene networks	[63,64]
Cell migration, invasion & metastasis	ABL1, ANXA3, FAK (PTK2)	Enhanced adhesion turnover, cytoskeletal remodeling, and EMT facilitate metastatic spread	Kinase inhibitors targeting migratory and invasive signaling pathways	[65,66]
Chromatin remodeling & mitotic stress	NEK2, VPS72	Chromosomal instability and altered chromatin dynamics promote tumor evolution and drug resistance	Mitotic kinase and chromatin-targeted therapies to restore genomic stability	[67,68]
Proteostasis & stress tolerance	YIF1B	Enhanced ER stress tolerance and protein folding capacity support tumor survival	Targeting ER stress pathways to induce tumor cell death	[69]

### 3.1 Major Pathways and Molecular Mechanisms

#### 3.1.1 Receptor Tyrosine Kinase (RTK) Driven Pathways

**Figure 3:**
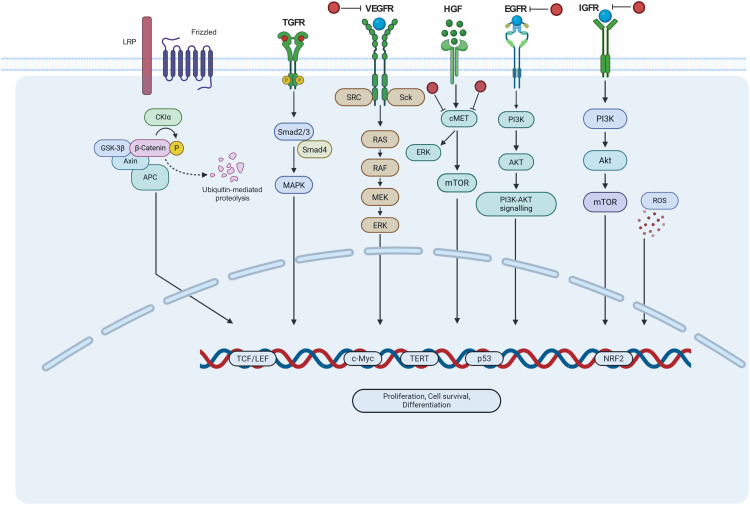
HCC cell survival proliferation and differentiation occur through the activation of interconnected signaling pathways. Inhibitors (red circles) (e.g., lenvatinib, sorafenib, atezolizumab, bevacizumab) target interdependent intracellular signaling pathways such as EGFR, FGFR, IGFR, TGFR, and VEGFR can alter several targets to exhibit anti-HCC effects (Figure generated by Biorender.com; https://www.biorender.com/). LRP: Low Density Lipoprotein Receptor-Related Protein; Frizzled: Frizzled Class Receptor; CKIα: Casein Kinase 1 Alpha; GSK-3β: Glycogen Synthase Kinase 3 Beta; Axin: Axis Inhibition Protein; APC: Adenomatous Polyposis Coli; TCF/LEF: T-Cell Factor/Lymphoid Enhancer-Binding Factor; TGFR: Transforming Growth Factor-Beta Receptor; Smad2/3: Mothers Against Decapentaplegic Homolog 2/3; Smad4: Mothers Against Decapentaplegic Homolog 4; MAPK: Mitogen-Activated Protein Kinase; SRC: Proto-Oncogene Tyrosine-Protein Kinase Src; RAS: Rat Sarcoma Viral Oncogene Homolog; RAF: Rapidly Accelerated Fibrosarcoma Kinase; MEK: MAPK/ERK Kinase; ERK: Extracellular Signal-Regulated Kinase; VEGFR: Vascular Endothelial Growth Factor Receptor; HGF: Hepatocyte Growth Factor; c-MET: Mesenchymal-Epithelial Transition Factor Receptor; PI3K: Phosphoinositide 3-Kinase; AKT: Protein Kinase; mTOR: Mechanistic Target of Rapamycin; EGFR: Epidermal Growth Factor Receptor; IGFR: Insulin-Like Growth Factor Receptor; ROS: Reactive Oxygen Species; NRF2: Nuclear Factor Erythroid 2-Related Factor 2; p53: Tumor Protein p53; TERT: Telomerase Reverse Transcriptase; c-Myc: MYC Proto-Oncogene.

The molecular pathogenesis of HCC is initiated by aberrant activation of signaling networks, with receptor tyrosine kinases (RTKs) representing a critical upstream driver. RTKs such as epidermal growth factor receptor (EGFR), fibroblast growth factor receptor (FGFR), hepatocyte growth factor receptor (c-MET), and vascular endothelial growth factor receptor 8(VEGFR) are frequently dysregulated through sustained growth factor stimulation (Fig. 3). This hyperactivation promotes malignant transformation and drives tumor progression. Downstream of RTKs, two key oncogenic cascades dominate: the RAS/RAF/MEK/ERK pathway, which regulates proliferation, angiogenesis, and metastasis, and the PI3K/AKT/mTOR pathway, which enhances proliferation, invasion, metabolic reprogramming (via the Warburg effect) and is associated with aggressive tumor biology and adverse clinical outcomes.

#### 3.1.2 Wnt/β-Catenin Pathway and Other Pathways

Dysregulation of Wnt/β-catenin signaling, found in ~30%–40% of HCC cases, contributes to transcriptional reprogramming, tumor progression, and the establishment of an immune cold phenotype. The β-catenin pathway can modulate the immune microenvironment, promoting immune evasion. The nuclear accumulation of β-catenin, the pathway’s key effector, is strongly associated with activating mutations in CTNNB1 (encoding β-catenin) and loss-of-function mutations in AXIN1 [33]. Other pathways, such as JAK/STAT and Hippo, also play significant roles. The JAK/STAT pathway, specifically through the activation of STAT3 by pro-inflammatory cytokines, influences gene expression and the behavior of activated hepatic stellate cells, which are crucial in liver fibrosis and injury response [34]. The Hippo pathway participates in HCC by regulating cell proliferation, apoptosis, and stem cell self-renewal. While these pathways may appear to function independently, a fundamental principle of HCC pathogenesis is their profound interconnectedness. The primary pathways are not isolated entities but are organized in a clear hierarchical manner, with RTKs serving as a central command-and-control hub that initiates multiple, parallel downstream cascades [35]. The binding of a single ligand to a receptor can trigger both major proliferation and survival pathways simultaneously, illustrating a powerful, convergent point of dysregulation where a singular insult can have broad, pleiotropic effects [36,37].

#### 3.1.3 PI3K/AKT/mTOR and Wnt/β-Catenin Pathways

Extensive functional interplay exists between the PI3K/AKT/mTOR and Wnt/β-catenin signaling pathways, both of which are central to cellular growth and metabolism. A key enzyme is glycogen synthase kinase-3β (GSK-3β), a component of the β-catenin destruction complex. Under physiological conditions, GSK-3β phosphorylates β-catenin, targeting it for degradation. However, PI3K/AKT signaling inhibits GSK-3β, stabilizing β-catenin and promoting its nuclear accumulation, where it interacts with LEF/TCF transcription factors to activate oncogenic gene expression. This direct linkage allows PI3K/AKT signaling to reinforce Wnt activity, promoting stemness, metastasis, and resistance to therapy (28).

#### 3.1.4 Notch Pathway and Wnt/β-Catenin Signaling

The Notch signaling pathway, highly conserved and frequently upregulated in HCC, adds another dimension to tumor pathogenesis. Ligand induced cleavage of Notch receptors releases the Notch intracellular domain (NICD), which translocates to the nucleus to regulate gene transcription. Importantly, NICD acts synergistically with Wnt/β-catenin signaling, amplifying transcriptional outputs that drive proliferation, tumor progression, and therapeutic resistance.

#### 3.1.5 Oncofetal Markers: Glypican-3 (GPC3) and Alpha-Fetoprotein (AFP)

Oncofetal proteins play pivotal roles in HCC pathogenesis and clinical management. GPC3, a cell surface heparan sulfate proteoglycan, is overexpressed in ~70% of HCC cases and correlates with poor prognosis [38]. Functionally, it promotes tumor growth by modulating FGF2 activity and activating canonical Wnt signaling. AFP, a fetal glycoprotein that undergoes aberrantly re-expressed during hepatocarcinogenesis. While it is widely utilized as a gold-standard diagnostic and prognostic serum biomarker, AFP also functions as a direct mediator of oncogenesis. It contributes to a multifaceted pathogenic network by stimulating angiogenesis, enhancing metastatic potential, and further suppressing anti-tumor immune responses.

#### 3.1.6 TERT Reactivation and Cell Cycle Dysregulation

Telomerase reverse transcriptase (TERT) promoter mutations are among the earliest and most prevalent genomic alterations in HCC, enabling replicative immortality. Concurrent dysfunction of cell-cycle regulators, including CDK4/6, CCND1, and RB1, further accelerates uncontrolled proliferation. These alterations establish cell-cycle disruption as a central oncogenic dependency in HCC.

Collectively, these findings highlight how RTK driven cascades, Wnt/β-catenin, PI3K/AKT/mTOR, Notch, telomerase reactivation, and oncofetal proteins converge into an interconnected oncogenic network. Their extensive crosstalk and feedback loops amplify malignant signaling, fuel immune evasion, and reinforce a pro-malignant phenotype. This complexity underscores both the challenges and opportunities in HCC therapy, pointing toward biomarker guided strategies, rational pathway specific inhibitors, and combinatorial regimens as essential approaches to improving clinical outcomes.

### 3.2 Tumor Microenvironment (TME) and Immune Checkpoints

**Figure 4:**
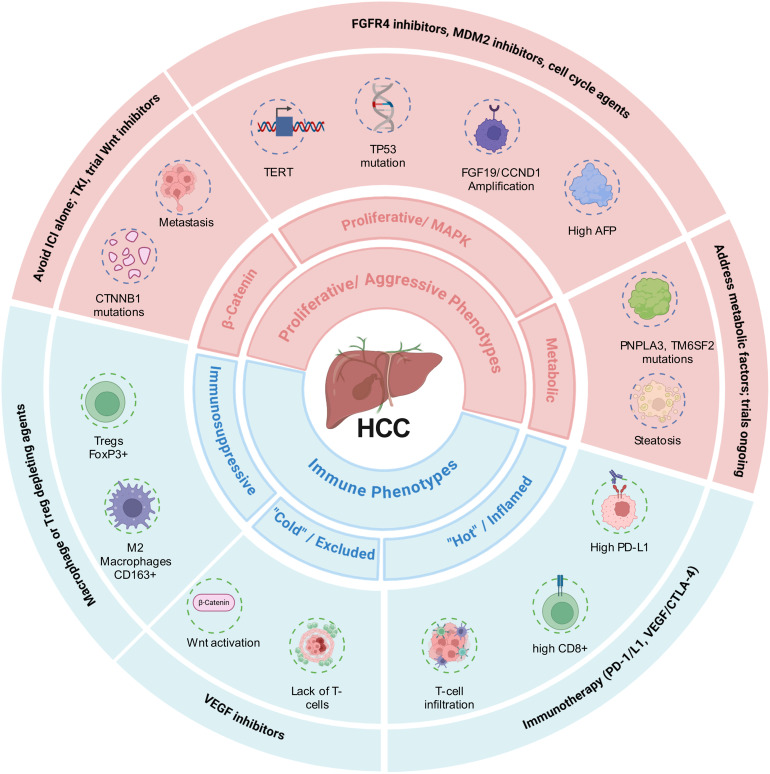
A multi-layered, precision medicine framework for HCC classification and therapy. The molecular and immune classification is composed of proliferative (Wnt/β-Catenin activated, proliferative/MAPK activated and metabolic syndrome related) and immune phenotypes (Inflamed, Excluded, and Immunosuppressive). Key biomarkers (e.g., CTNNB1 mutation, high PD-L1/CD8^+^ T cell density, FGF19 amplification) are the specific evidence used for definitive subtyping. Associated therapeutic strategies provides subtype-specific treatment recommendations: immunotherapy (anti-PD-1/L1/anti-VEGF/CTLA-4) is prioritized for immune hot/suppressive subtypes, targeted therapies (FGFR4 inhibitors, MDM2 inhibitors) for proliferative subtypes, and novel combinations (locoregional and immunotherapy) are proposed to convert immune cold tumors to hot phenotypes. HCC: Hepatocellular Carcinoma; TKI: Tyrosine kinase inhibitor; ICI: Immune Checkpoint Inhibitor; MAPK: Mitogen-Activated Protein Kinase; CTNNB1: Catenin Beta 1; TERT: Telomerase Reverse Transcriptase; TP53: Tumor Protein p53; FGF19: Fibroblast Growth Factor 19; CCND1: Cyclin D1; AFP: Alpha-Fetoprotein; PNPLA3: Patatin-Like Phospholipase Domain-Containing Protein 3; TM6SF2: Transmembrane 6 Superfamily Member 2; PD-L1: Programmed Death-Ligand 1; VEGFR: Vascular Endothelial Growth Factor Receptor; PD-1: Programmed Cell Death Protein 1; CTLA-4: Cytotoxic T-Lymphocyte-Associated Protein 4; FoxP3: Forkhead Box P3; Tregs: Regulatory T Cells; CD163: Cluster of Differentiation 163; CD8^+^: Cluster of Differentiation 8–Positive T Cells; Wnt: Wingless/Integrated Signaling Pathway; FGFR4: Fibroblast Growth Factor Receptor 4; MDM2: Mouse Double Minute 2 Homolog.

The TME is a highly dynamic ecosystem composed of malignant cells, stroma, extracellular matrix (ECM), and immune populations that actively orchestrates tumor survival, angiogenesis, and immune evasion (Fig. 4). Central to this immunosuppressive landscape is the upregulation of inhibitory checkpoints that induce T cell exhaustion and promote immune tolerance. The PD-1/PD-L1 axis is the most clinically validated pathway; tumor cells exploit PD-L1 expression to deactivate effector T cells directly within the TME (Fig. 4). CTLA-4 (Cytotoxic T-Lymphocyte-Associated protein 4) functions as a critical immune brake primarily during the early priming phase of T cell activation in the lymph nodes, distinct from the peripheral activity of PD-1. LAG-3 impairs CD4^+^ T cell activation via MHC-II trans-endocytosis and enhances Treg-mediated suppression [39]. TIM-3 receptor, expressed on exhausted CD8^+^ T cells, interacts with galectin-9 to inhibit interferon-γ secretion and drive macrophages toward a pro-tumorigenic, immunosuppressive phenotype.

### 3.3 Metabolic Reprogramming and Stress Adaptation

Metabolic plasticity is a defining feature of HCC, reflecting both tumor-intrinsic alterations and adaptation to the diseased liver microenvironment. HCC cells exhibit enhanced glycolysis, altered lipid metabolism, increased glutamine dependency, and dysregulated bile acid signaling [40]. In MASLD/NASH related HCC, lipid accumulation, oxidative stress, and chronic inflammation further complicate tumor metabolism and immune responses. These metabolic states can directly influence sensitivity to systemic therapies, including immunotherapy, underscoring the need for etiology aware treatment strategies [41].

### 3.4 Epigenetic Regulation and Non-Coding RNAs

Aberrant DNA methylation silences tumor suppressor genes such as CDKN2A, while deregulated histone modifications, alter chromatin accessibility and oncogenic transcriptional programs. These changes not only promote tumor initiation but also blunt antitumor immunity by repressing immune-stimulatory gene expression. Emerging data highlighted a mechanosensitive checkpoint Osr2 recruits HDAC3 to suppress cytotoxic T cell gene programs, directly connecting biomechanical stress with immune exhaustion [33].

## 4 Emerging Combination Strategies in HCC

The marked molecular heterogeneity and adaptive capacity of HCC impose fundamental limitations on monotherapy-based treatment paradigms. Clinical resistance frequently emerges through compensatory pathway activation, immune escape, and metabolic or phenotypic plasticity, resulting in transient or incomplete responses. Consequently, contemporary HCC management has shifted toward rational combination strategies designed to simultaneously target complementary oncogenic processes, remodel the TME, and enhance the durability of therapeutic responses. Building on the molecular vulnerabilities, this section focuses on how these targets are therapeutically integrated to overcome resistance and improve clinical outcomes.

### 4.1 Integration of Kinase Inhibitors and Immune-Based Therapies

**Figure 5:**
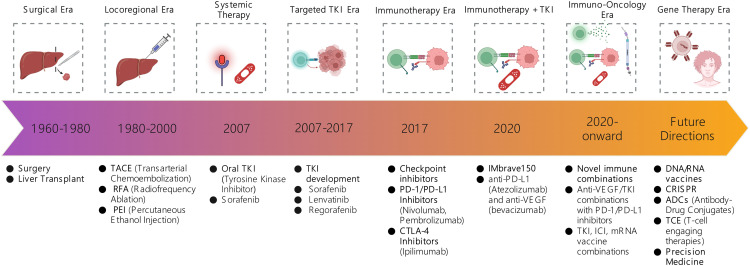
The Evolution of HCC management: A timeline from locoregional intervention to immunotherapy driven systemic care. ICI: Immune checkpoint inhibitor; CRISPR: Clustered Regularly Interspaced Short Palindromic Repeats; PD-1: Programmed Cell Death Protein 1; PD-L1: Programmed Death-Ligand 1; CTLA-4: Cytotoxic T-Lymphocyte-Associated Protein 4; VEGF: Vascular Endothelial Growth Factor; anti-PD-1: Anti-Programmed Cell Death Protein 1; anti-PD-L1: Anti-Programmed Death-Ligand 1; anti-VEGF: Anti-Vascular Endothelial Growth Factor.

Tyrosine kinase inhibitors (TKIs) were the first systemic agents to demonstrate survival benefit in advanced HCC; however, their efficacy is limited by rapid adaptive resistance. In parallel, ICIs have transformed HCC treatment but exhibit modest response rates when used alone, largely due to an immunosuppressive TME. These complementary limitations have driven the development of TKI-ICI combination regimens, which now form the backbone of first-line systemic therapy (Table 4). The mechanistic rationale underlying this approach lies in the ability of anti-angiogenic TKIs to normalize tumor vasculature, reduce hypoxia, and attenuate immunosuppressive cytokine signaling, thereby facilitating immune cell infiltration and function. When combined with ICI, this vascular and immunological reprogramming enhances T cell-mediated anti-tumor responses and mitigates intrinsic immune resistance (Fig. 5). Clinical validation of this strategy was achieved with the IMbrave150 trial, which established atezolizumab plus bevacizumab as the global first-line standard for unresectable HCC (can’t be removed by surgery). Beyond this regimen, multiple combinations pairing ICI with multi-targeted TKI are under active investigation, reflecting a sustained effort to refine efficacy while balancing toxicity profiles.

**Table 4:** Mechanism-driven combination therapy strategies in hepatocellular carcinoma (HCC).

Combination Strategy Class	Therapeutic Components	Mechanistic Rationale	Representative Agents	Ref.
Dual immune checkpoint blockade	PD-1/PD-L1 + CTLA-4 inhibitors	CTLA-4 blockade enhances T-cell priming in lymphoid organs, while PD-1/PD-L1 inhibition sustains effector T-cell activity and tumor infiltration, resulting in synergistic immune activation	Nivolumab, Ipilimumab, Pembrolizumab, Atezolizumab	[73]
Anti-angiogenic TKI + immunotherapy	VEGF/FGFR inhibitors + PD-1/PD-L1 blockade	TKIs normalize tumor vasculature, reduce immunosuppressive signaling, and enhance lymphocyte infiltration, thereby improving immune checkpoint inhibitor efficacy	Atezolizumab, Bevacizumab, Nivolumab, Erdafitinib	[70]
Multi-target TKI combinations	FGFR + VEGF or c-MET inhibitors	Simultaneous inhibition of angiogenesis and oncogenic growth signaling limits compensatory pathway activation and tumor adaptability	Erdafitinib, Crizotinib, Cabozantinib	[74]
Chemotherapy + immunotherapy	Cytotoxic agents + PD-1/PD-L1 blockade	Chemotherapy induces immunogenic cell death and antigen release, priming the immune system, while ICI sustain anti-tumor immune responses	Cisplatin, Doxorubicin, Nivolumab, Pembrolizumab	[75]
Immunotherapy + autophagy inhibition	PD-1/PD-L1 blockade + autophagy inhibitors	Autophagy inhibition disrupts tumor stress-adaptation mechanisms, enhancing immune-mediated tumor cell killing	Nivolumab, Pembrolizumab, Hydroxychloroquine	[76]
Chemotherapy + TKI combinations	Cytotoxic agents + VEGF/c-MET inhibitors	TKIs suppress angiogenesis and growth signaling, while chemotherapy directly induces tumor cell death, producing synergistic cytotoxicity	Crizotinib, Cabozantinib, Doxorubicin, Gemcitabine	[77]
TKI + immune modulators	FGFR/c-MET inhibitors + TGF-β inhibitors	TGF-β blockade reverses immune suppression within the TME while TKIs inhibit tumor proliferation and angiogenesis	Erdafitinib, Galunisertib, Crizotinib	[78]
Targeted therapy + lncRNA modulation	TKIs + lncRNA inhibitors	lncRNA targeting disrupts transcriptional programs linked to invasion and chemoresistance, enhancing sensitivity to targeted agents	Crizotinib, Erdafitinib, HOTAIR inhibitors, MALAT1 inhibitors	[79]
TKI + mTOR pathway inhibition	VEGF/c-MET inhibitors + mTOR inhibitors	Dual inhibition blocks angiogenesis and PI3K/Akt/mTOR-driven tumor growth, overcoming pathway redundancy	Everolimus, Temsirolimus, Bevacizumab, Crizotinib	[80]
Triplet strategies (chemotherapy + autophagy inhibition + immunotherapy)	Cytotoxic drugs + autophagy inhibitors + PD-1 blockade	Chemotherapy induces tumor cell death, autophagy inhibition prevents adaptive survival, and immunotherapy amplifies tumor-specific immune responses	Cisplatin, Doxorubicin, Hydroxychloroquine, Nivolumab	[81]

Note: FGFR: Fibroblast Growth Factor Receptor; c-MET: Mesenchymal-Epithelial Transition Factor (Hepatocyte Growth Factor Receptor); TGF-β: Transforming Growth Factor Beta; TME: Tumor Microenvironment; lncRNA: Long Non-Coding RNA; mTOR: Mechanistic Target of Rapamycin; PI3K: Phosphoinositide 3-Kinase; Akt: Protein Kinase.

#### 4.1.1 TKI-ICI Combinations

Multiple clinical trials are evaluating the combination of ICI with TKI that possess anti-angiogenic activity. These regimens aim to exploit synergistic effects by concurrently inhibiting oncogenic signaling, suppressing angiogenesis, and reinvigorating anti-tumor immunity. Clinical studies involving lenvatinib plus pembrolizumab, cabozantinib plus atezolizumab, and similar combinations has demonstrated encouraging objective response rates and progression-free survival benefits, supporting continued development in both frontline and later-line settings (Table 5) [70]. Importantly, the success of these combinations has underscored the need for biomarker-driven patient selection, as not all tumors derive equal benefit from immune-angiogenic modulation [71].

**Table 5:** Key clinical trials in advanced HCC combination therapy.

Trial Name	Regimen	Comparator	Median OS	Median PFS	ORR	Key Outcome
IMbrave150	Atezolizumab /Bevacizumab	Sorafenib	19.2 mo	6.8 mo	30%	New 1L standard; 8% CR rate
HIMALAYA	Durvalumab /Tremelimumab	Sorafenib	16.4 mo	3.8 mo	20.1%	Dual-ICI; 30.7% 3-yr OS
LEAP-012	TACE/Pembrolizu mab/Lenvatinib	TACE alone	Immature	Improved		Improved PFS in intermediate HCC

Note: OS: Overall Survival; PFS: Progression-Free Survival; ORR: Objective Response Rate; mo: Months; CR: Complete Response; 1L: First-Line (therapy); ICI: Immune Checkpoint Inhibitor; yr: Year; TACE: Transarterial Chemoembolization; HCC: Hepatocellular Carcinoma.

#### 4.1.2 ICI-ICI Combinations

An alternative strategy to enhance immune activation involves the simultaneous blockade of multiple immune checkpoints. Dual inhibition of PD-1/PD-L1 and CTLA-4 targets distinct phases of the T cell response, with CTLA-4 blockade enhancing early T cell priming and PD-1/PD-L1 inhibition sustaining effector function within the tumor [72]. This approach has demonstrated clinical efficacy in advanced HCC, with durable responses observed in subsets of patients. While immune-related adverse events remain a concern, optimized dosing schedules and patient stratification have improved the therapeutic index of dual ICI regimens, positioning them as viable options for selected patients.

#### 4.1.3 ICI and MAPK Pathway Inhibitors Combinations

Given the frequent activation of the MAPK signaling cascade in HCC, combinations incorporating MEK or RAF inhibitors with ICI have emerged as a strategy to counteract immune resistance driven by oncogenic signaling. MAPK pathway inhibition may reduce tumor-induced immune suppression and enhance antigen presentation, thereby sensitizing tumors to immune checkpoint blockade. Although still largely in early-phase clinical development, this approach represents a rational extension of pathway-informed combination therapy, particularly for tumors exhibiting MAPK-driven resistance phenotypes.

#### 4.1.4 Locoregional Therapy (LRT) and Systemic Therapy Combinations

Locoregional therapies (LRT), including transarterial chemoembolization (TACE), radiofrequency ablation (RFA), and radioembolization, remain central to HCC management across disease stages. Increasing evidence suggests that LRT can induce immunogenic tumor cell death, releasing tumor antigens that prime systemic immune responses [82]. Combining LRT with systemic agents particularly ICI and anti-angiogenic therapies leverages this immunogenic effect to enhance systemic disease control. Ongoing trials evaluating TACE or Y90 radioembolization in combination with ICI and TKI have reported improvements in progression-free survival, supporting the concept of locoregional-systemic synergy in intermediate-stage disease [83].

#### 4.1.5 RNA Targeted Therapeutics

Despite advances in targeted and immune-based therapies, directly targeting core genetic drivers of HCC remains challenging. RNA-based therapeutics, including small interfering RNAs (siRNAs) and microRNAs (miRNAs), offer a powerful modality to modulate oncogenic gene expression post-transcriptionally [84,85]. Preclinical studies have demonstrated that multi-target RNA strategies can suppress multiple oncogenic pathways simultaneously, mirroring the rationale of multi-drug therapy. When integrated with TKI or ICI, RNA-based agents have the potential to sensitize tumors, overcome resistance, and enhance therapeutic durability. Advances in delivery technologies particularly lipid nanoparticles and hepatocyte-targeted conjugates have significantly improved the translational feasibility of these approaches.

### 4.2 Future Directions: Toward Tri-Modal and Integrated Therapy

The future of HCC treatment lies in integrated, multi-modal strategies that combine molecular targeting, immune modulation, and microenvironmental reprogramming. A conceptual framework involves a sequenced approach:

- An RNA agent (e.g., siRNA) silences a primary resistance driver (e.g., CTNNB1).
- A TKI/anti-angiogenic agent remodels the TME.
- An ICI is administered to unleash a potent and durable immune response against the now-sensitized tumor.

While such regimens offer substantial promise, their complexity introduces challenges related to toxicity, treatment sequencing, and cost. Addressing these barriers will require the development of predictive biomarkers, optimized dosing strategies, and adaptive trial designs. Ultimately, the successful implementation of integrated combination therapies will depend on precision medicine approaches that align therapeutic intensity with tumor biology and patient-specific risk profiles. The vast majority of trials combine ICI, TKI, anti-VEGF agents, and locoregional therapy (Table 6).

**Table 6:** HCC combination therapy and rationale for combination.

Therapeutic Modality	Primary Role	Biological Rationale	Targets	Clinical Integration
Immune Checkpoint Inhibitors (ICI)	Immune priming and durable tumor control	Reinvigorate exhausted cytotoxic T cells and restore antitumor immunity; most effective in immune-inflamed TME	PD-1/PD-L1 (nivolumab, pembrolizumab, atezolizumab, durvalumab); CTLA-4 (ipilimumab, tremelimumab)	First-line systemic therapy; backbone of combination regimens; adaptive escalation in immune-responsive tumors
Tyrosine Kinase Inhibitors (TKIs)/Anti-Angiogenics	Resistance modulation and vascular normalization	Inhibit compensatory oncogenic signaling (VEGF, FGFR, c-MET) and normalize abnormal vasculature to enhance immune infiltration	Sorafenib, lenvatinib, cabozantinib, regorafenib; bevacizumab	Combined with ICI; sequencing or switching upon molecular or radiologic evidence of resistance
RNA-Based Therapeutics (siRNA, miRNA)	Targeting core genetic drivers and resistance mechanisms	Post-transcriptional modulation enables direct targeting of undruggable oncogenic drivers	siRNA (TERT, CTNNB1, NET1, EMS1); miRNAs (miR-122, miR-21)	Emerging modality; integrated into adaptive frameworks guided by molecular profiling
Epigenetic Modulators	Immune sensitization and transcriptional reprogramming	Reverse epigenetic silencing of tumor suppressor and immune-related genes	DNMT inhibitors (guadecitabine); HDAC inhibitors (panobinostat, chidamide)	Combination with ICI to overcome immune resistance
Locoregional Therapies (LRT)	Tumor debulking and immunogenic priming	Induce immunogenic cell death and tumor antigen release, enhancing systemic immune responses	TACE, RFA, microwave ablation, Y-90 radioembolization	Combined with systemic therapy in intermediate-stage or oligoprogressive disease
AI-Guided Therapeutic Sequencing	Dynamic treatment optimization	Integrates longitudinal molecular, imaging, and clinical data to anticipate resistance and guide therapy adaptation	ML/DL models, reinforcement learning frameworks	Decision-support tool for adaptive precision oncology
Liquid Biopsy-Driven Surveillance	Early detection of resistance and minimal residual disease	Circulating tumor DNA/RNA reflects real-time tumor evolution before radiologic progression	ctDNA, cfRNA, circulating miRNAs	Guides early treatment modification and sequencing decisions

Note: ICI: Immune Checkpoint Inhibitor; TME: Tumor Microenvironment; PD-1: Programmed Cell Death Protein 1; PD-L1: Programmed Death-Ligand 1; CTLA-4: Cytotoxic T-Lymphocyte-Associated Protein 4; TKIs: Tyrosine Kinase Inhibitors; VEGF: Vascular Endothelial Growth Factor; FGFR: Fibroblast Growth Factor Receptor; c-MET: Mesenchymal–Epithelial Transition Factor; siRNA: Small Interfering RNA; miRNA: MicroRNA; TERT: Telomerase Reverse Transcriptase; CTNNB1: Catenin Beta 1; NET1: Neuroepithelial Cell Transforming 1; EMS1: Excess Microsporocytes 1; DNMT: DNA Methyltransferase; HDAC: Histone Deacetylase; LRT: Locoregional Therapy; TACE: Transarterial Chemoembolization; RFA: Radiofrequency Ablation; Y-90: Yttrium-90 Radioembolization; ctDNA: Circulating Tumor DNA; cfRNA: Cell-Free RNA.

A significant number of trials are investigating the combinations in the neoadjuvant (pre-surgery) and adjuvant (post-curative treatment) settings to reduce recurrence rates (Table 6). Many trials are specifically focused on treating patients who have progressed on the current first-line standard of care. Emerging agents target new pathways such as TIGIT (Tiragolumab), LAG-3 (Relatlimab), TIM-3 (Cobolimab), CTLA-4 with enhanced Fc function (BMS-986218), and cellular therapies (CAR-T, CAR-Macrophages). The integration and timing of TACE, TARE (transarterial radioembolization), SBRT (Stereotactic Body Radiation Therapy), and HAIC (Hepatic Artery Infusion Chemotherapy) with systemic therapy is a major area of active investigation (Table 7).

**Table 7:** HCC ongoing clinical trials highlighting the novel agents and their targets.

NCT Number	Phase	Treatment Setting	Therapeutic	Novel Agent(s)	Target(s)	Trial Design	Line of Therapy
**First-Line Systemic Therapy**							
NCT05904886 (IMbrave152)	III	Systemic	Anti-PD-L1 + Anti-VEGF + Anti-TIGIT	Tiragolumab	TIGIT	Randomized, Quadruple-Blind	1st
NCT05883644	III	Systemic	Anti-PD-L1 + Anti-CTLA-4 (STRIDE regimen)	Durvalumab + Tremelimumab	PD-L1, CTLA-4	Single-Group Assignment	1st
NCT04039607	III	Systemic	Anti-PD-1 + Anti-CTLA-4	Nivolumab + Ipilimumab	PD-1, CTLA-4	Randomized vs. SOC	1st
NCT03755791 (COSMIC-312)	III	Systemic	TKI + Anti-PD-L1 vs. TKI	Cabozantinib + Atezolizumab	MET, VEGFR, AXL, PD-L1	Randomized vs. Sorafenib	1st
NCT04194775	III	Systemic	Anti-PD-1 + TKI	Nofazinlimab (CS1003) + Lenvatinib	PD-1, Multi-Kinase	Randomized, Quadruple-Blind	1st
**Second-Line Systemic Therapy**							
NCT07138885	II	Systemic /Locoregional	Dual ICI (Anti-PD-1/CTLA-4) + HAIC/TACE	QL1706	PD-1, CTLA-4	Single-Arm	2nd (Post TKI-IO)
NCT05199285	II	Systemic	Anti-PD-1 + Anti-CTLA-4	Nivolumab + Ipilimumab	PD-1, CTLA-4	Single-Arm	2nd (Post Atezo + Bev)
NCT06138769	II	Systemic	TKI	Lenvatinib	Multi-Kinase	Single-Arm	2nd (Post Atezo + Bev)
NCT05178043	II	Systemic	Anti-PD-1 + Anti-GPC3	Nivolumab + GT90001	PD-1, GPC3	Single-Arm	2nd (Post ICI)
**Neoadjuvant/Adjuvant Therapy**							
NCT05908786	Ib/II	Neoadjuvant	Various immuno-oncology (IO) Combinations	Atezo + Bev +/− Tiragolumab, Tobemstomig	PD-L1, VEGF, TIGIT, etc.	Randomized Platform	Pre-Surgery
NCT05389527	II	Neoadjuvant	Anti-PD-1 + TKI	Pembrolizumab + Lenvatinib	PD-1, Multi-Kinase	Single-Arm	Pre-Surgery
NCT04102098 (IMbrave050)	III	Adjuvant	Anti-PD-L1 + Anti-VEGF	Atezolizumab + Bevacizumab	PD-L1, VEGF	Randomized vs. Surveillance	Post-Curative
NCT03867084 (KEYNOTE-937)	III	Adjuvant	Anti-PD-1	Pembrolizumab	PD-1	Randomized, Double-Blind	Post-Curative
**Combination with Locoregional Therapy**							
NCT04246177 (LEAP-012)	III	Unresectable	TACE/Anti-PD-1/TKI	Pembrolizumab /Lenvatinib	PD-1, Multi-Kinase	Randomized, Quadruple-Blind	1st
NCT05301842	III	Locoregional	TACE + Anti-PD-L1 +/− Anti-CTLA-4 +/− TKI	Durvalumab /Tremelimumab /Lenvatinib	PD-L1, CTLA-4, Multi-Kinase	Randomized	1st
NCT04712643	III	Locoregional	TACE + Anti-PD-L1 + Anti-VEGF	Atezolizumab /Bevacizumab	PD-L1, VEGF	Randomized vs. TACE	1st
**Basket/Bucket Trials**							
NCT06638931 (ANTARES)	II	Systemic (Agnostic)	Anti-PD-1	Nivolumab	PD-1	Basket Trial	Later Line
NCT05293496	I	Systemic	BiTE + Anti-PD-1/CTLA-4	Vobramitamab duocarmazine/Lorigerlimab	B7-H3, PD-1/CTLA-4	Basket Trial	Later Line
NCT03860272	I	Systemic	Fc-enhanced Anti-CTLA-4/Anti-PD-1	Botensilimab /Balstilimab	CTLA-4 (Fc-enhanced), PD-1	Basket Trial	Later Line

Note: TIGIT: T Cell Immunoreceptor with Ig and ITIM domains; PD-L1: Programmed Death-Ligand 1; CTLA-4: Cytotoxic T Lymphocyte Associated Protein 4; MET: MET Receptor Tyrosine Kinase; VEGFR: Vascular Endothelial Growth Factor Receptor; AXL: AXL Receptor Tyrosine Kinase; GPC3: Glypican 3; B7-H3: B7 Homolog 3 Protein; BiTE: Bi-Specific T Cell Engager.

### 4.3 Etiology-Specific Molecular Signatures and Therapeutic Implications

HCC arises from diverse etiologies can reshape tumor biology and therapeutic responsiveness (Table 8). HBV-associated HCC is characterized by viral DNA integration, HBx-mediated malfunction of oncogenic signaling pathways, *TERT* promoter and *TP53* mutations, AKT pathway activation, and epigenetic silencing of tumor suppressor genes, generating a relatively “inflamed” TME with T-cell infiltration and often enhanced responsiveness to ICI [86]. In contrast, HCV-associated HCC arises mainly through chronic inflammation, oxidative stress, and immune disruption rather than direct genomic integration, with frequent CTNNB1 and AXIN1 alterations promoting a WNT/β-catenin driven “immune-excluded” TME and resistance to anti-PD-1 therapy. MASLD/NASH-related HCC exhibits pronounced metabolic reprogramming, lipotoxicity, IL-6/JAK/STAT pathway activation, immune exhaustion, and characteristic genetic variants such as PNPLA3, TM6SF2, and MBOAT7, resulting in a metabolically inflamed, immunosuppressive TME that reduces ICI efficacy but highlights potential vulnerabilities to metabolic and cytokine-targeted therapies [87,88]. Similarly, ALD-associated HCC shows overlapping features with MASLD-HCC, including IL-6/JAK/STAT activation and immunosuppressive TME shaped by chronic alcoholic injury, alongside acetaldehyde-induced DNA damage, oxidative stress, and aberrant methylation patterns that accelerate hepatocarcinogenesis. Finally, aflatoxin-related HCC is defined by a high-frequency *TP53* R249S mutation caused by aflatoxin B1 exposure, often synergizing with HBV infection, and generating a genotoxin-driven TME with vulnerabilities in DNA damage repair and cell cycle control [89].

**Table 8:** Molecular and therapeutic distinctions across major HCC etiological subtypes.

Etiological Subtype	Molecular Drivers/Features	Immune Microenvironment (TME) Characteristics	Therapeutic Implications/Vulnerabilities
HBV-associated HCC	High prevalence of TERT promoter and TP53 mutations. HBV DNA integration, AKT pathway activation	An “inflamed” or “hot” TME; T-cell infiltration is often present, driven by chronic viral inflammation	Often shows a more favorable response to ICI/Anti-PD-1 compared to non-viral HCC, making the TME permissive to immune activation
HCV-associated HCC	High frequency of CTNNB1 mutations (WNT/β-catenin pathway activation) and AXIN1 alterations	WNT/β-catenin activation tends to promote an “immune-excluded” or “cold” TME phenotype, with reduced T-cell infiltration	Predictive Resistance to ICI: CTNNB1-mutated tumors are largely unresponsive to anti-PD-1 monotherapy
MASLD/NASH-associated HCC	Lower frequency of classic oncogenic mutations. Aberrant metabolic/lipid pathways. Frequent activation of IL-6/JAK/STAT	Highly immunosuppressive TME. Characterized by metabolic inflammation and lipid-overloaded macrophages	Reduced Sensitivity to ICI: The immunometabolic TME often leads to resistance. Vulnerabilities: Targeting metabolic pathways or the IL-6/JAK/STAT axis
Alcohol-associated HCC (ALD-HCC)	High rates of TERT promoter mutations. Steatohepatitic HCC (SH-HCC) subtype. Shares activation of the IL-6/JAK/STAT signaling pathway with MASLD-HCC	TME is shaped by chronic alcoholic injury, typically leading to an immunosuppressive state	Therapeutic strategies may focus on overcoming chronic inflammation and the suppressive TME components, similar to MASLD-HCC
Aflatoxin-related HCC	Molecular Fingerprint: A specific, high-frequency TP53 R249S mutation (G:C → T:A transversion) resulting from Aflatoxin B1-DNA adducts	The TME is defined by the specific genotoxic insult, often co-occurring with HBV	Targeting DDR (DNA Damage Repair): The specific TP53 mutation suggests vulnerabilities in cell cycle control and DDR pathways

Note: HBV: Hepatitis B Virus; HCC: Hepatocellular Carcinoma; TERT: Telomerase Reverse Transcriptase; TP53: Tumor Protein p53; ICI: Immune Checkpoint Inhibitor; PD-1: Programmed Cell Death Protein 1; HCV: Hepatitis C Virus; CTNNB1: Catenin Beta 1; WNT: Wingless/Integrated Signaling Pathway; AXIN1: Axis Inhibition Protein 1; MASLD: Metabolic Dysfunction-Associated Steatotic Liver Disease; NASH: Nonalcoholic Steatohepatitis; IL-6: Interleukin 6; JAK: Janus Kinase; STAT: Signal Transducer and Activator of Transcription; ALD: Alcohol-Associated Liver Disease; SH-HCC: Steatohepatitic Hepatocellular Carcinoma; DDR: DNA Damage Response.

While ICI, particularly combinations such as atezolizumab plus bevacizumab, have redefined first-line therapy for advanced HCC, critical gaps remain that limit their universal efficacy [90]. Notably, chronic viral HCC tends to be “hot” and inflamed, favoring ICI responsiveness, whereas MASH/NASH-HCC often exhibits a “cold” microenvironment, potentially rendering ICI ineffective or even detrimental; however, pivotal trials have largely failed to stratify patients by etiology [89]. Moreover, current studies exclude patients with compromised liver function (Child-Pugh B/C), leaving their safety and efficacy profiles uncertain. High-frequency alterations in the TERT promoter (up to 60%) and Wnt/β-catenin pathway (CTNNB1 mutations, up to 30%) remain largely undruggable, highlighting the need for next-generation targeted therapies, including small-molecule inhibitors or gene-editing approaches, potentially in combination with ICI, to achieve durable responses across HCC subtypes [91]. Collectively, these etiological subtypes illustrate that HCC is not a uniform disease entity; integrating molecular, immunological, and metabolic profiling into precision oncology frameworks enables etiology-stratified biomarker development, individualized immunotherapeutic and metabolic interventions, and rationalized clinical trial design [91].

## 5 Advancing Precision Oncology in Hepatocellular Carcinoma: Multi-Omics, AI, and Spatial Technologies

The therapeutic landscape of HCC is undergoing a fundamental transition toward precision oncology, a paradigm that tailors interventions to the molecular, spatial, and immunological architecture of individual tumors. This shift reflects the recognition that HCC is not a single disease entity but a spectrum of biologically distinct malignancies shaped by diverse etiologies, evolutionary trajectories, and TME states. The convergence of multi-omics profiling, spatial transcriptomics (ST) and artificial intelligence (AI) now enables a systems-level understanding of HCC that surpasses traditional clinic-pathologic stratification. Collectively, these technologies support earlier diagnosis, robust prediction of therapeutic response, and rational design of individualized treatment strategies.

### 5.1 Multi-Omics Data Resources for Precision HCC Research

Comprehensive multi-omics profiling is essential for capturing the molecular complexity of HCC. Large-scale public consortia have generated foundational datasets integrating genomics, transcriptomics, epigenomics, proteomics, and clinical annotation. The Cancer Genome Atlas-Liver Hepatocellular Carcinoma (TCGA-LIHC) cohort provides matched genomic, transcriptomic, epigenomic, and proteomic data for approximately 360–400 primary HCC tumors, predominantly derived from patients with viral hepatitis (HBV/HCV) or metabolic liver disease (NAFLD/MASH). Complementing TCGA, the International Cancer Genome Consortium (ICGC) incorporates ethnically and etiologically diverse cohorts from multiple geographic regions, expanding the generalizability of molecular discoveries, as highlighted in Table 9. Transcriptomic datasets from the Gene Expression Omnibus (GEO) and European Nucleotide Archive (ENA) further support validation studies, particularly for TME characterization and drug resistance mechanisms. Proteogenomic data from the Clinical Proteomic Tumor Analysis Consortium (CPTAC) provide direct insights into functional protein states and post-translational modifications linked to prognosis and treatment response.

**Table 9:** Multi-omics data sources and landscape characterization.

Omics Layer	Data Type	Sequencing Platform	Sample Size	Key Clinical Insights	Data Source
Genomics	Somatic Mutations, CNVs	Illumina HiSeq 2000/2500 Whole Exome Sequencing (WES)	~360–400 unique HCC tumors	Etiology (HBV, HCV, Alcohol, Other), Age, Sex, Tumor Grade, Vascular Invasion, TNM Stage	TCGA-LIHC
Transcr- iptomics	Gene Expression	Illumina RNA-Seq	~360–400 matched tumors and adjacent non-tumor tissues	Liver Cirrhosis Status, AFP Level, Treatment Response Data	TCGA-LIHC
Proteomics	Protein Abundance, PTMs	Mass Spectrometry	~150–200 subsets of the TCGA/ICGC cohorts	Overall Survival (OS), Progression-Free Survival (PFS), Recurrence Status	TCGA-LIHC/CPTAC
Epigenomics	DNA Methylation	Illumina HumanMethylation450/EPIC BeadChip	~360–400 matched tumor/normal pairs	Geographic Origin, Aflatoxin Exposure	TCGA-LIHC

Note: CNV: Copy Number Variation; PTM: Post-Translational Modification; HBV: Hepatitis B Virus; HCV: Hepatitis C Virus; AFP: Alpha-Fetoprotein; TCGA-LIHC: The Cancer Genome Atlas-Liver Hepatocellular Carcinoma. Clinical Proteomic Tumor Analysis Consortium (CPTAC); URL: https://portal.gdc.cancer.gov/projects/TCGA-LIHC.

### 5.2 Deciphering HCC Complexity through Integrated Multi-Omics Analysis

Single-layer molecular analyses are insufficient to explain HCC pathogenesis or therapeutic resistance. Integrated multi-omics approaches overcome this limitation by combining genomics, transcriptomics, proteomics, epigenomics, and metabolomics to construct a comprehensive molecular portrait of the tumor. Genomic analyses reveal recurrent alterations in oncogenic and tumor suppressor pathways, including Wnt/β-catenin, PI3K/AKT/mTOR, MAPK/ERK, and *TP53*, which inform patient stratification for targeted therapies and immunotherapy. Transcriptomic profiling captures dynamic gene expression programs associated with proliferation, immune exclusion, metastasis, and drug resistance, while also identifying non-coding RNAs (lncRNAs and miRNAs) as emerging biomarkers and therapeutic targets. Proteomics provides a functional readout of tumor biology by quantifying signaling proteins, immune checkpoint expression, and angiogenic mediators, often outperforming mRNA-based predictors. Epigenomic and metabolomic analyses further uncover regulatory and metabolic rewiring, including aberrant DNA methylation and enhanced glycolysis, fatty acid oxidation, and glutaminolysis.

**Figure 6:**
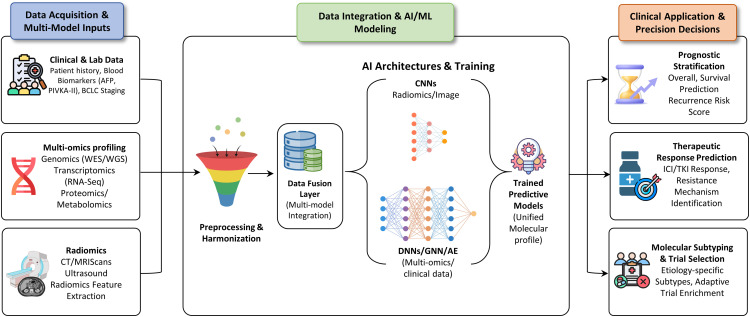
The multi-omics to AI pipeline for HCC precision oncology. Conceptual framework illustrating the integration of heterogeneous clinical, molecular, and imaging data through advanced AI/ML architectures to generate actionable, personalized clinical guidance for HCC management. BCLC: Barcelona Clinic Liver Cancer; WES: Whole Exome Sequencing; WGS: Whole Genome Sequencing; CT: Computed Tomography; MRI: Magnetic Resonance Imaging; CNN: Convolutional Neural Network; DNN: Deep Neural Network; GNN: Graph Neural Network; AE: Autoencoder; ICI: Immune Checkpoint Inhibitor; TKI: Tyrosine Kinase Inhibitor; AFP: Alpha-Fetoprotein.

Crucially, integrated multi-omics signatures outperform single biomarkers in predicting prognosis and therapeutic response. Systems-level analyses have identified robust prognostic programs, including PANoptosis-related gene signatures, as well as actionable vulnerabilities in treatment-refractory HCC, underscoring the translational value of multi-modal integration (Fig. 6) [69].

### 5.3 Artificial Intelligence Architectures for Precision Oncology in HCC

The high dimensionality and heterogeneity of multi-omics, imaging, and clinical datasets necessitate advanced AI and machine-learning (ML) frameworks capable of extracting clinically actionable insights. Deep learning architectures, particularly convolutional neural networks (CNN) for imaging and deep neural networks (DNNs) for multimodal data fusion now form the computational backbone of precision oncology in HCC (Table 10).

**Table 10:** Artificial intelligence architectures in HCC.

Application	AI/ML Methodology	Clinical Impact	Ref.
Prognosis	CNN/Deep Learning & integrated clinical data	Predict progression-free and overall survival/stratify recurrence risk	[92]
Diagnosis	CNN/Radiomics (CT/MRI)	Non-invasive prediction of high-risk tumor features (e.g., MVI)	[93]
Therapy Prediction	XGBoost/Ensemble ML	Predict response and survival benefit from TKI + ICI therapy	[94,95]

Note: CT: Computed Tomography; MRI: Magnetic Resonance Imaging; ICI: Immune Checkpoint Inhibitor; TKI: Tyrosine Kinase Inhibitor; MVI: Microvascular Invasion.

#### 5.3.1 AI-Driven Radiomics and Imaging Analytics

Radiomics leverages AI to extract quantitative features from contrast-enhanced CT and MRI that are imperceptible to visual inspection. CNN-based radiomics models are most extensively validated for predicting microvascular invasion (MVI), recurrence risk, and treatment response. Three-dimensional CNN architectures trained across arterial, portal venous, and delayed phases implicitly learn features related to tumor margin irregularity, intra-tumoral heterogeneity, and peritumoral enhancement. Meta-analyses report pooled AUC values exceeding 0.85 for MVI prediction and recurrence risk stratification, supporting radiomics as a reliable non-invasive adjunct [96–99].

#### 5.3.2 AI for Multi-Omics Integration and Therapeutic Response Prediction

DNNs excel at integrating heterogeneous data streams, including genomic alterations (e.g., *CTNNB1*, *TERT*, *TP53*), transcriptomic immune signatures, proteomic markers, imaging features, and clinical variables. Modality-specific embedding layers followed by fusion architectures enable synergistic learning across data types and consistently outperform single-modality models. Autoencoders (AE) are commonly used to compress high-dimensional transcriptomic data into biologically meaningful latent representations. In HCC, such models have demonstrated strong performance in predicting response to ICI based therapies and locoregional treatments, with pooled AUROC values approaching 0.89 in internal validation and ~0.81 in external cohorts (Table 10) [100,101].

### 5.4 Clinical Performance, Validation, and Generalizability

AI models in HCC have achieved clinically meaningful performance across diagnostic, prognostic, and therapeutic domains (Table 11). Radiomics-based classifiers differentiate HCC from benign or cirrhotic nodules with pooled AUCs of approximately 0.86, while survival prediction models achieve C-indices ranging from 0.73 to 0.78 in external validation cohorts [86,102,103]. Integrated multi-omics models further enhance survival prediction, reporting 1-year survival AUCs approaching 0.98 in TCGA-based analyses and improved long-term prognostic accuracy when incorporating key driver genes *(FBN1, MAP1B)* [104,105].

**Table 11:** Clinical performance & validation of AI/ML models in HCC management.

Task	Data Type	Model Architecture	Validation Cohort (*n*)	Performance Metric	Key Findings
Prediction of Post-Liver Transplant (LT) Recurrence	Clinical variables (Tumor Diameter, AFP, Age, PIVKA-II, portal vein thrombosis (PVT)	DNN /DeepSurv	Multicenter Validation (*n* = 563 total)	C-index: 0.75 (Validation Cohort)	Outperformed established criteria (Milan, UCSF, Kyoto), enabling improved candidate selection, survival analysis
Prediction of 5-year Overall Survival (OS) in Large HCC (LHCC)	Clinical variables (NLR, Platelet count, Tumor Size, BCLC Stage)	Gradient Boosting Machine (GBM)	Validation Cohort (*n* = 457)	AUC: 0.75 (5-year OS)	Stratified LHCC into distinct prognostic groups, aiding in personalized follow-up intensity
Prediction of Immunotherapy Response	Radiomics (CT-derived features tumor and surrounding liver)	Support Vector Machine (SVM)/Naïve Bayes	Cross-Validation (*n* = 353)	Accuracy: 86% (Combined Model)	Developed a non-invasive, pre-treatment biomarker to identify responders to ICI
HCC Risk in Chronic Hepatitis B HBV-CHB Patients	Clinical/Laboratory variables (Age, platelet count, Albumin, HBV DNA)	Random Forest (RF)	External Validation (*n* = 1937)	AUC: 0.872 (Validation Cohort)	Superior prediction of 5-year HCC development in patients receiving antiviral therapy, guiding surveillance strategies

Note: CT: Computed Tomography; DNN: Deep Neural Network; HBV: Hepatitis B Virus; NLR: Neutrophil-to-lymphocyte ratio; BCLC: Barcelona Clinic Liver Cancer staging system.

Despite these advances, generalizability remains a major barrier. Models trained in HBV-predominant populations frequently underperform in MASLD-dominant cohorts, reflecting true biological heterogeneity rather than technical bias [86]. Addressing this challenge requires federated learning, multinational collaboration, and harmonization of imaging protocols, radiomics pipelines, and omics preprocessing.

### 5.5 Toward Adaptive Precision Oncology in HCC

Static treatment algorithms are insufficient to address tumor evolution and microenvironmental plasticity. Adaptive precision oncology reframes treatment as a dynamic, data-driven process in which therapy evolves in response to longitudinal molecular and immunological changes. This framework integrates baseline molecular stratification, AI-guided therapy selection, serial liquid biopsy surveillance, and dynamic therapeutic adaptation.

**Figure 7:**
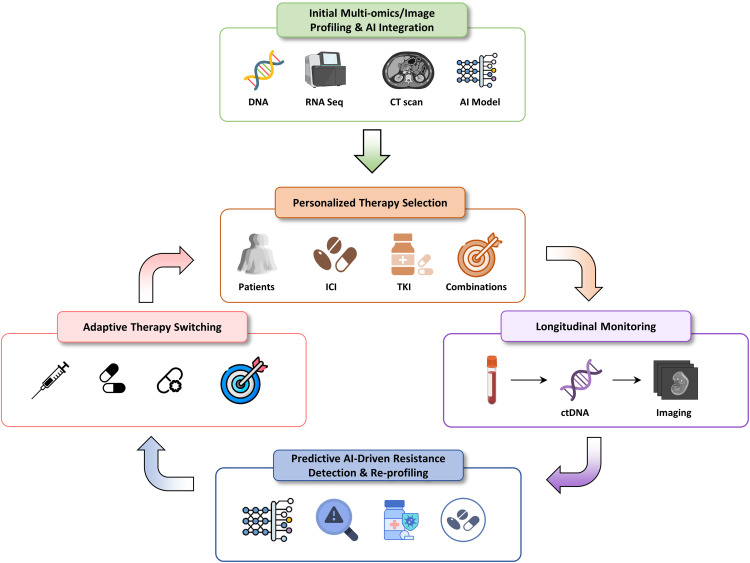
Adaptive precision oncology framework in HCC. The cyclic process of adaptive precision oncology, where initial profiling guides therapy, and continuous AI-powered monitoring of tumor evolution dictates real-time treatment modifications to overcome resistance. RNA-Seq: RNA Sequencing; CT: Computed Tomography; AI: Artificial Intelligence; ICI: Immune Checkpoint Inhibitor; TKI: Tyrosine Kinase Inhibitor; ctDNA: Circulating Tumor DNA.

AI-driven platforms integrating multi-omics, radiomics, and clinical data can anticipate resistance mechanisms (often preceding radiographic progression) and recommend timely therapeutic modification. Reinforcement learning (RL) models and graph-based frameworks further enable simulation of tumor evolution under different treatment pressures, informing optimal sequencing strategies [86]. Collectively, these approaches redefine HCC management as an anticipatory and personalized process, with AI serving as the analytical backbone for next-generation precision oncology (Fig. 7).

Algorithms such as YOLOv8 and frameworks (MONAI) extract quantitative features from medical images to enable earlier HCC detection, non-invasive characterization of tumor grade and vascular invasion, and more accurate monitoring of disease progression (Table 12). Furthermore, AI tools (Visiopharm and Aiforia) provide quantitative analysis of the TME from digital pathology images, elucidating immune cell infiltration patterns and spatial relationships that are critical for predicting immunotherapy outcomes. The development of generative AI models promises to create sophisticated *in silico* simulations of tumor evolution. By integrating the full spectrum of omics data, these models aim to reconstruct the dynamic interplay within the TME, predict responses to novel interventions, and ultimately guide the development of truly personalized, adaptive therapeutic strategies for every HCC patient.

**Table 12:** Key models and tools of Artificial Intelligence (AI) in HCC.

Area	AI Model	Clinical Metric	Clinical Relevance	Tools/Utility
Imaging & Volumetrics	YOLO8, U-Net, CNN	AUC: 0.91–0.98 for lesion detection; C-index: 0.82 for FLR survival	Automates BCLC staging; predicts post-hepatectomy liver failure (PHLF) via functional volumetrics.	Aiforia aiforia.com, Quibim QP Liver quibim.com, MONAI monai.io Perspectum’s Hepatica (FDA-cleared)
Virtual Biopsy	ResNet-50, XGBoost, Radiomic-Clinic Fusion	AUC: 0.88–0.93 MVI prediction	Identifies MVI pre-operatively to guide wider surgical margins vs. anatomical resection	Clinically Validated (e.g., Jiang et al. multicenter models), GE Healthcare
Histopathology	DL for Whole-Slide Image (WSI), HCCnet, MesoNet	C-index: 0.75–0.78 (Recurrence-free survival)	Predicts recurrence following transplant/resection directly from H&E; identifies high-risk adjuvant candidates	Owkin owkin.com/(HCCnet) (EMA Support), PathAI pathai.com (AISight Dx) (FDA-cleared)
PathGenomics	Vision Transformers (ViT), HE2RNA models	AUC: 0.78 (CTNNB1); AUC: 0.81 (*TP53*)	Economic surrogate for NGS; identifies “Immune-Cold” (CTNNB1 mut) patients unlikely to respond to IO-VEGF	*In Silico* Research
Dynamic Prognosis	RNNs, LSTMs, LSTM-D (Temporal)	UC: 0.85–0.90 (5-year HCC risk in CHB/MASLD).	Longitudinal monitoring of HBV/MASLD patients; outperforms static models (e.g., mPAGE-B) in predicting onset.	Research Models
Drug Discovery & synergy	GNNs, Reinforcement Learning (RL)	AUC: 0.68–0.74 (*In vitro* synergy prediction)	Discovery of bypass inhibitors (e.g., MET/FGFR4) to overcome Sorafenib resistance.	DeepChem, Insilico Medicine
Multi-Omics Integration	DELFI (Fragmentomics), TECPI	AUC: 0.92–0.96 (Early detection)	Early-stage HCC screening using cell-free DNA (cfDNA) fragmentation patterns	DELFI delfi.bio, Tempus tempus.com (Alcyone)
Tumor Microenvironment	Spatial AI models, Graph Convolutional Nets	C-index: 0.72–0.75 (IO Response)	Analyzes spatial proximity of CD8^+^ T-cells to tumor cells to predict immunotherapy response.	Aiforia aiforia.com, Visiopharm visiopharm.com

Note: CNN: Convolutional Neural Network; AUC: Area Under the Curve; C-index: Concordance Index; FLR: Future Liver Remnant; BCLC: Barcelona Clinic Liver Cancer; PHLF: Post-Hepatectomy Liver Failure; FDA: U.S. Food and Drug Administration; XGBoost: Extreme Gradient Boosting; MVI: Microvascular Invasion; DL: Deep Learning; WSI: Whole-Slide Image; H&E: Hematoxylin and Eosin; EMA: European Medicines Agency; ViT: Vision Transformer; CTNNB1: Catenin Beta 1; TP53: Tumor Protein p53; NGS: Next-Generation Sequencing; RNN: Recurrent Neural Network; LSTM: Long Short-Term Memory; LSTM-DLSTM-D: Long Short-Term Memory with Decay; HCC: Hepatocellular Carcinoma; CHB: Chronic Hepatitis B; MASLD: Metabolic Dysfunction–Associated Steatotic Liver Disease; GNN: Graph Neural Network; RL: Reinforcement Learning; MET: Mesenchymal–Epithelial Transition Factor; FGFR4: Fibroblast Growth Factor Receptor 4; cfDNA: Cell-Free DNA; DELFI: DNA Evaluation of Fragments for Early Interception; IO: Immuno-Oncology.

### 5.6 Spatial Transcriptomics (ST): Mapping TME for the Therapeutic Precision

While multi-omics profiling reveals what molecular alterations are present in a tumor, it often homogenizes the sample, obscuring the critical architectural context of where these alterations occur. ST addresses this fundamental limitation by mapping gene expression with cellular-to-subcellular resolution directly within the native tissue architecture [106]. This transformative technology provides an unprecedented view of the TME, enabling the deconstruction of the intricate cellular ecosystems, signaling networks, and metabolic niches that collectively govern HCC progression, immune evasion, and therapeutic response [107]. In HCC, a malignancy defined by profound intra-tumoral heterogeneity, ST is particularly powerful. It moves beyond bulk analysis to delineate distinct tumor subclones coexisting within a single lesion, each with unique molecular features. For instance, it can reveal regions characterized by high expression of angiogenic markers (e.g., VEGF) adjacent to zones expressing immune checkpoint proteins (e.g., PD-L1). This granular mapping is essential for understanding the functional consequences of tumor evolution and for designing therapies that can address the tumor’s multifaceted biology. Furthermore, this technology is revolutionizing our understanding of the immunosuppressive landscape of HCC. By spatially resolving the locations and gene expression profiles of diverse cell populations, researchers can precisely map the “battlegrounds” within the TME. This includes identifying immunosuppressive hubs enriched with regulatory T cells (Tregs), myeloid derived suppressor cells (MDSCs), tumor associated macrophages (TAMs), and visualizing the physical barriers that lead to immune exclusion zones, where cytotoxic T cells are prevented from infiltrating the tumor core. Understanding these spatial dynamics is critical for developing next-generation immunotherapies, such as CAR-T cell therapies or novel checkpoint inhibitors, designed to reprogram the TME and overcome resistance.

The synergy between ST and AI is essential for unlocking the full potential of this data rich technology. The sheer complexity of spatial datasets necessitates sophisticated computational methods for analysis. AI algorithms, particularly deep learning models like convolutional neural networks (CNNs), are being deployed to automate the identification of histological features, quantify the density and spatial distribution of tumor infiltrating lymphocytes (TILs), and define recurrent cellular neighborhoods that correlate with clinical outcomes. Moreover, AI is enabling the development of 3D spatial reconstructions of the TME, providing multi-dimensional insights into the interactions between tumor, stromal, and immune cells. This powerful combination is not only elucidating fundamental mechanisms of immune evasion but is also identifying novel, spatially defined biomarkers and therapeutic targets. Integrating these spatial insights with multi-omics data has moved us towards a new era of precision oncology where therapeutic strategies are tailored not only to the molecular profile of a tumor but also to its unique spatial architecture.

*Practical Constraints on Spatial Transcriptomics (ST)*

While techniques such as ST and multi-modal AI modeling represent the scientific apex of precision oncology, their clinical implementation faces significant practical barriers. Despite its revolutionary power to map cellular interactions within the TME, the clinical utility of ST is currently hampered by high per-sample cost, low throughput compared to bulk sequencing, and the lack of standardized computational pipelines. These factors restrict its use primarily to specialized research institutions, delaying its deployment as a routine clinical tool. Although many AI models show high performance *in silico*, integrating them into diverse clinical settings is challenging. Deployment requires significant IT infrastructure investment, data harmonization efforts across multiple imaging systems, and ongoing regulatory approval and maintenance. This high initial and operational cost often creates a disparity, limiting the availability of these advanced predictive tools to large academic medical centers, thereby potentially increasing existing health disparities. The foundational data for multi-omics and ST requires high-quality tissue. HCC often presents in patients with cirrhosis, making repeated, invasive tumor biopsies risky and limiting the amount of available tissue. This constraint forces reliance on less precise liquid biopsy methods or radiomics, highlighting a fundamental physical limitation of data acquisition in this disease setting.

## 6 Translational Insights and Future Directions

Rapid advances in molecular profiling, spatial biology, and computational analytics are refurbishing the conceptual framework of HCC research. The central challenge now lies in converting these biological insights into clinically actionable strategies that enable durable, patient-specific benefit. Bridging discovery science with therapeutic implementation will define the next phase of progress in HCC management.

### 6.1 The Translation Gap and Preclinical Limitations

Despite an expanding catalog of oncogenic drivers, immune regulators, epigenetic modifiers, and metabolic dependencies in HCC, only a limited number of discoveries have translated into approved therapies. This translational gap largely reflects the limitations of preclinical models (like standard cell lines and basic CDX models) that inadequately recapitulate the complexity of human TME, including tumor heterogeneity, immune contexture, vascular remodeling, and underlying liver dysfunction [108].

Successful examples, such as the clinical validation of immune checkpoint inhibition combined with anti-angiogenic therapy, illustrate the importance of aligning mechanistic rationale with clinically representative models and biomarker-driven trial design [109]. Future translational research must prioritize advanced preclinical systems such as patient-derived organoids, spatially resolved co-culture models, and humanized mouse platforms that faithfully mimic the TME and chronic liver disease context [110]. These models are essential for improving therapeutic predictability and reducing late-stage clinical attrition.

### 6.2 Personalized Therapeutic Strategies and Resistance Mitigation

The intrinsic molecular diversity of HCC and the inevitability of adaptive resistance necessitate a transition from uniform treatment algorithms to individualized therapeutic strategies. Limited tissue availability and spatial heterogeneity remain major barriers to precision medicine; however, the expanding use of liquid biopsy offers a scalable solution for non-invasive, longitudinal tumor profiling. Circulating tumor DNA and RNA analyses enable real-time monitoring of clonal evolution, emergent resistance mechanisms, and immune dynamics, supporting adaptive treatment modification [111]. The clinical utility of liquid biopsy will depend on the development of robust biomarkers capable of guiding therapeutic selection and response assessment. AI-driven analytical frameworks are poised to play a pivotal role by integrating multi-omics, imaging, and clinical data to enhance signal detection, reduce noise, and identify composite molecular signatures that define clinically relevant HCC subtypes. Such approaches move beyond single biomarkers toward multidimensional predictors with greater clinical robustness.

Overcoming therapeutic resistance remains a critical objective. As a single pathway rarely drives resistance in HCC; rather, compensatory signaling networks enable tumor persistence under selective pressure. This complexity underscores the need for rationally designed combination strategies that target shared regulatory nodes or convergent vulnerabilities. Emerging RNA-targeted therapeutics provide a promising orthogonal modality by enabling direct modulation of oncogenic transcripts and resistance-associated regulators [112,113].

### 6.3 PANoptosis and Multi-Target Therapeutic Convergence

PANoptosis is recognized as a bridge between inflammatory signaling and cell death. Its imbalance can suppress antigen presentation, alter cytokine production, and create an immunosuppressive TME. Multi-omics analyses have identified PANoptosis associated regulators and gene signatures linked to aggressive, treatment-refractory HCC phenotypes. Therapeutically activating PANoptosis has the potential to convert “cold” tumors into “hot” immunogenic lesions, thereby enhancing tumor immunogenicity, sensitize tumors to immune checkpoint blockade, and synergize with kinase inhibitors [114]. Integrating PANoptosis associated biomarkers into immunotherapy stratification models may refine patient selection and help mitigate the heterogeneity in treatment outcomes. This strategy exemplifies how systems-level biological insights can inform mechanism based therapeutic innovation.

### 6.4 Etiology and AI-Guided Precision Oncology

Precision oncology in HCC therefore requires a shift from histology- and stage-based treatment toward etiology- and molecularly stratified decision-making. AI and ML are essential enablers of this transition. By integrating etiological background, multi-omics profiles, imaging features, and longitudinal clinical data, AI models can stratify patients into biologically coherent subgroups and predict optimal therapeutic sequencing [88]. Future clinical trials must adopt stratified designs that evaluate therapeutic efficacy within defined etiological-molecular subtypes, rather than treating HCC as a homogeneous entity [95]. Such trial paradigms will be critical for maximizing the benefit of emerging systemic and combination therapies.

## 7 Conclusions

Hepatocellular carcinoma remains a major global health challenge, characterized by high mortality, biological complexity, and limited long-term therapeutic success in advanced disease. Although combination immunotherapy has redefined the treatment landscape and improved outcomes for selected patients, durable benefit remains elusive for many, largely due to profound tumor heterogeneity and adaptive resistance. The shifting epidemiology of HCC from viral hepatitis toward metabolic and alcohol-associated etiologies further underscores the need for updated screening strategies, tailored therapeutic approaches, and renewed pharmaceutical investment. The future of HCC management lies in the convergence of multi-omics profiling, artificial intelligence, and spatially resolved technologies to enable biologically informed, adaptive treatment strategies. The therapeutic paradigm is evolving from monotherapy toward intelligently sequenced, multi-modal regimens that integrate immunotherapy, kinase inhibition, and RNA-targeted approaches. Achieving this vision will require rigorous biomarker development, innovative clinical trial designs, and close integration of computational models into clinical decision-making. By accelerating the translation of systems-level insights into personalized interventions, precision oncology has the potential to transform HCC from a largely lethal disease into a manageable and ultimately curable condition.

## Data Availability

Data sharing is not applicable to this article.
